# Neurocognitive Mechanisms Underlying Social Atypicalities in Autism: Weak Amygdala’s Emotional Modulation Hypothesis

**DOI:** 10.3389/fpsyt.2020.00864

**Published:** 2020-09-04

**Authors:** Wataru Sato, Shota Uono, Takanori Kochiyama

**Affiliations:** ^1^ Psychological Process Team, BZP, RIKEN, Kyoto, Japan; ^2^ Organization for Promoting Neurodevelopmental Disorder Research, Kyoto, Japan; ^3^ Department of Developmental Disorders, National Institute of Mental Health, National Center of Neurology and Psychiatry, Tokyo, Japan; ^4^ Brain Activity Imaging Center, ATR-Promotions, Kyoto, Japan

**Keywords:** amygdala, autism spectrum disorder (ASD), emotion, emotional facial expression, functional magnetic resonance imaging (fMRI)

## Abstract

Autism spectrum disorder (ASD) is a neurodevelopmental condition associated with atypicalities in social interaction. Although psychological and neuroimaging studies have revealed divergent impairments in psychological processes (e.g., emotion and perception) and neural activity (e.g., amygdala, superior temporal sulcus, and inferior frontal gyrus) related to the processing of social stimuli, it remains difficult to integrate these findings. In an effort to resolve this issue, we review our psychological and functional magnetic resonance imaging (fMRI) findings and present a hypothetical neurocognitive model. Our psychological study showed that emotional modulation of reflexive joint attention is impaired in individuals with ASD. Our fMRI study showed that modulation from the amygdala to the neocortex during observation of dynamic facial expressions is reduced in the ASD group. Based on these findings and other evidence, we hypothesize that weak modulation from the amygdala to the neocortex—through which emotion rapidly modulates various types of perceptual, cognitive, and motor processing functions—underlies the social atypicalities in individuals with ASD.

## Introduction

Autism spectrum disorder (ASD) is a neurodevelopmental condition associated with atypicalities in reciprocal social interaction (1). Characteristic symptoms of social atypicalities in individuals with ASD include difficulty in perception and recognition of faces and facial expressions (2–4), eye gaze (5–7), action (8–10), and the inner states (11–13) of other individuals.

The neurocognitive mechanisms underlying the social atypicalities of ASD remain elusive. Psychological studies have shown that various types of social cognitive processes are atypical in individuals with ASD compared with those of typically developing (TD) individuals; these atypicalities include emotional, perceptual, cognitive, and motor processing (14). In ASD groups, functional neuroimaging studies also revealed atypical brain activity associated with processing of social stimuli in multiple brain regions, including the amygdala, fusiform gyrus, superior temporal sulcus, and inferior frontal gyrus (15–17). However, it remains difficult to integrate these divergent psychological and neuroscientific findings to explain the social atypicalities of ASD.

In this article, we briefly review our psychological and neuroscientific findings and present a neurocognitive model seeking to further the understanding of this issue. Our psychological experiment revealed that individuals with ASD exhibit impaired rapid emotional modulation of attentional shift triggered by gaze. Our functional magnetic resonance imaging (fMRI) study demonstrated that individuals with ASD exhibit weak modulatory effects from the amygdala to the neocortical network during the observation of facial expressions. Based on these findings and other evidence, we speculate that weak modulation from the amygdala to the neocortex, *via* which emotion rapidly modulates various types of perceptual, cognitive, and motor processing functions, underlies the social atypicalities of individuals with ASD.

### Psychological Study of Emotional Modulation of Reflexive Joint Attention

In the first psychological experiment, we investigated the modulatory effect of emotion on reflexive joint attention (18).

Clinical observations have suggested that one of the most obvious social atypicalities in individuals with ASD is a deficit of joint attention with others (19). However, a number of experimental psychological studies found that individuals with ASD can exhibit intact reflexive joint attention using the cueing paradigm (20, 21). In an effort to resolve this discrepancy, some studies in TD individuals showed that reflexive joint attention can be positively modulated by emotional facial expressions, especially when presented dynamically (22–29), although the effects with static expressions are still debated (30). A developmental study showed that this emotional enhancement of joint attention starts by at least 7 years of age (28). Some studies found that higher autistic traits are associated with weaker emotional enhancement of reflexive joint attention (27, 31). Numerous studies reported that individuals with ASD are impaired in terms of processing emotions in facial expressions (2–4, 32), even at the rapid, unconscious processing stage (33); thus, we hypothesized that individuals with ASD would show impaired joint attention when gaze cues were combined with emotional facial expressions. However, direct evidence of this was lacking. Other studies testing the integration between gaze and emotional expressions in individuals with ASD using different paradigms have reported mixed findings (34–38).

We tested this hypothesis in adolescent/adult individuals with ASD and TD controls using a dynamic fearful gaze as the cue of the cueing paradigm.

#### Methods

We tested 11 high-functioning individuals with ASD (three women; mean ± SD age, 17.5 ± 6.5 years) and 11 age- and sex-matched TD individuals (three women; mean ± SD age, 19.5 ± 2.2 years). All participants in the ASD group had intelligence quotients (IQs) in the normal range and were diagnosed with Asperger’s disorder or pervasive developmental disorder not otherwise specified (PDD-NOS) with milder symptoms of Asperger’s disorder.

**Figure 1:**
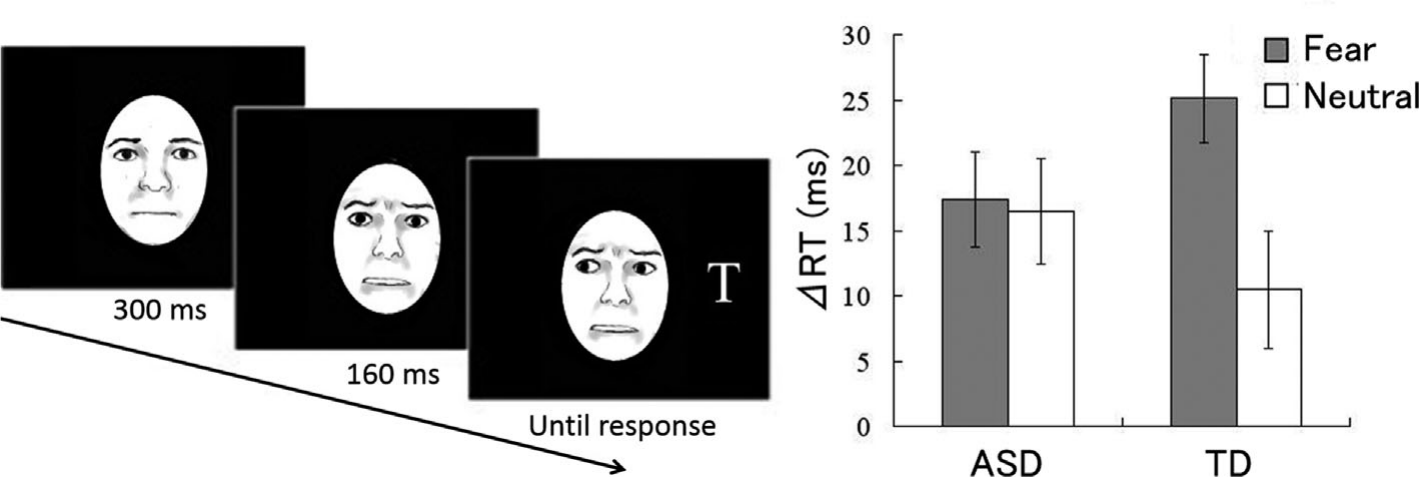
Study by Uono et al. (18). (Left) Illustration of dynamic fearful gaze. (Right) Mean (± standard error) reaction time differences between invalid and valid gaze conditions for autism spectrum disorder (ASD) and typically developing (TD) groups.

As cue stimuli, dynamic fearful and neutral facial expressions with changing gaze directions were presented (**Figure 1**); dynamic expressions were created from the photographs of neutral and fearful faces (39) using computer morphing. Stimuli were sequentially presented from neutral to 100% fearful under fearful gaze conditions. Under the neutral gaze condition, only the gaze direction was dynamically changed.

In each trial (**Figure 1**), after point fixation, a dynamic fearful or neutral facial cue with the eyes directed toward either the left or right was presented at the center of the monitor. Subsequently, a target letter “T” appeared in either the left or right visual field Participants were instructed to specify as quickly as possible whether the target appeared by pressing a button; the reaction times (RTs) of button pressing were measured. The participants were instructed that the cues were non-predictive of target appearance.

#### Results

The RT differences between valid and invalid conditions were used as indices of the gaze cueing effect. One-sample *t*-tests for RT differences (**Figure 1**) showed that the gaze cueing effects were significantly greater than zero in response to both fearful and neutral cues in both the ASD and TD groups. Emotion (fear, neutral) × group (ASD, TD) analysis of variance revealed a significant main effect of emotion and a significant interaction. Follow-up analyses of the interaction demonstrated that the gaze cueing effect was significantly greater in response to fearful cues than to neutral cues in the TD group, but not in the ASD group.

#### Discussion

Our data under the neutral condition confirmed that gaze cues triggered reflective joint attention in individuals with ASD as observed in previous studies.

More importantly, the gaze cueing effect was greater in response to fearful cues than to neutral cues only in the TD group. The results demonstrate that a dynamic emotional gaze does not facilitate reflexive joint attention in individuals with ASD. Our results may, at least in part, account for the discrepancy between clinical observations and experimental findings in terms of joint attention in individuals with ASD. In naturalistic communication, emotional gaze by other individuals provides important information regarding their evaluation of attended objects (40) and their inner mental state (41). Therefore, although TD individuals evidently show reflective joint attention with the facilitative effect of emotion, individuals with ASD may fail to show such emotional joint attention; this lack of attention may result in difficulty in social learning and social interaction.

Our results indicate that emotional modulation of attentional processing is impaired in individuals with ASD, although their reliability should be further confirmed due to the relatively small sample size and several possible confounding factors (e.g., age). Similar findings have been reported in other studies that used different experimental paradigms. For instance, one previous study using the visual search paradigm showed that the facilitated perception of happy facial expressions in TD individuals was lacking in individuals with ASD (42). When dynamic facial expressions with subtle emotions were presented and participants were instructed to match the image in the response panel to the final image of the stimulus, individuals with ASD were less likely to perceive exaggerated facial expressions than were TD individuals (43). Another study showed that automatic congruent facial responses to emotional facial expressions were reduced in individuals with ASD compared with TD individuals; however, basic facial motor functions were not impaired in individuals with ASD (44). These data suggest that emotional modulation of various types of perceptual, cognitive, and motor processing functions may be atypical in individuals with ASD.

### fMRI Study of the Amygdala-Neocortex Network During Facial Expression Processing

In the second fMRI experiment, we explored the functional neural networking patterns underlying atypical processing of dynamic facial expressions (45).

Multiple previous fMRI studies have evaluated the brain activities associated with atypical facial expression processing in individuals with ASD [e.g., (46)]. Although the results varied somewhat, most studies reported that observation of emotional facial expressions induced less activation in the ASD group than the TD group, in both subcortical regions (e.g., the amygdala) and neocortical regions (e.g., the fusiform gyrus, superior temporal sulcus, and inferior frontal gyrus). A considerable body of neuropsychological and neuroimaging evidence from TD individuals implies that these brain regions are related to specific social information processing, such as emotional evaluation in the amygdala (47), visual decoding of faces in the fusiform gyrus and superior temporal sulcus (48), and action-observation matching in the inferior frontal gyrus (49). These data suggest that reduced activity in these subcortical and neocortical regions may underlie atypical facial expression processing in individuals with ASD. Among these regions, the amygdala has long received much attention in ASD research based on divergent lines of evidence; for example, the amygdala works as a hub for brain regions associated with social perception and social affiliation (50), which are affected in individuals with ASD; amygdala-damaged monkeys show abnormal behavioral symptoms similar to ASD (51, 52); and the amygdala in individuals with ASD show structural regional abnormalities, both microscopic (53, 54) and macroscopic (55, 56), and connectivity abnormalities (57).

However, the coupling patterns between the subcortical and neocortical regions during observation of emotional facial expressions in individuals with ASD remain unclear. A previous study (58) investigated this issue in TD individuals by analyzing fMRI data while the participants observed dynamic facial expressions using dynamic causal modeling (DCM); this allows inferences to be drawn about the causal and directional effects among brain regions (59). The models compared included those considering a modulatory effect of dynamic expressions from the amygdala to the neocortical network, from the neocortical network to the amygdala, and a joint effect. The optimal model was that featuring a modulatory effect from the amygdala to the neocortical network. This result is compatible with anatomical findings in monkeys; specifically, the amygdala receives inputs *via* subcortical visual pathways that bypass the neocortical visual pathways (60), and it sends projections to many neocortical regions, including the visual and motor cortices (61). Based on these data and the above-described psychological findings on reduced emotional modulation of facial expression processing in individuals with ASD, we hypothesized that modulation of the neocortical network by the amygdala during observation of dynamic facial expressions may be reduced in individuals with ASD.

We tested this hypothesis in an fMRI experiment with ASD and TD groups. The participants passively observed dynamic facial expressions of anger and happiness and dynamic randomized mosaic images (control stimuli). Following analysis of group differences in regional brain activity, we performed DCM and compared models featuring modulation of dynamic expressions from the amygdala to the neocortical network, from the neocortical network to the amygdala, and a joint effect. We predicted that the first model would optimally explain the between-group differences.

#### Methods

We tested 31 high-functioning individuals with ASD (nine women; mean ± SD age, 27.2 ± 8.5 years) and 31 age- and sex-matched TD individuals (nine women; mean ± SD age, 24.2 ± 1.0 years). All participants in the ASD group had IQs in the normal range and were diagnosed with Asperger’s disorder or PDD-NOS with milder symptoms of Asperger’s disorder.

**Figure 2:**
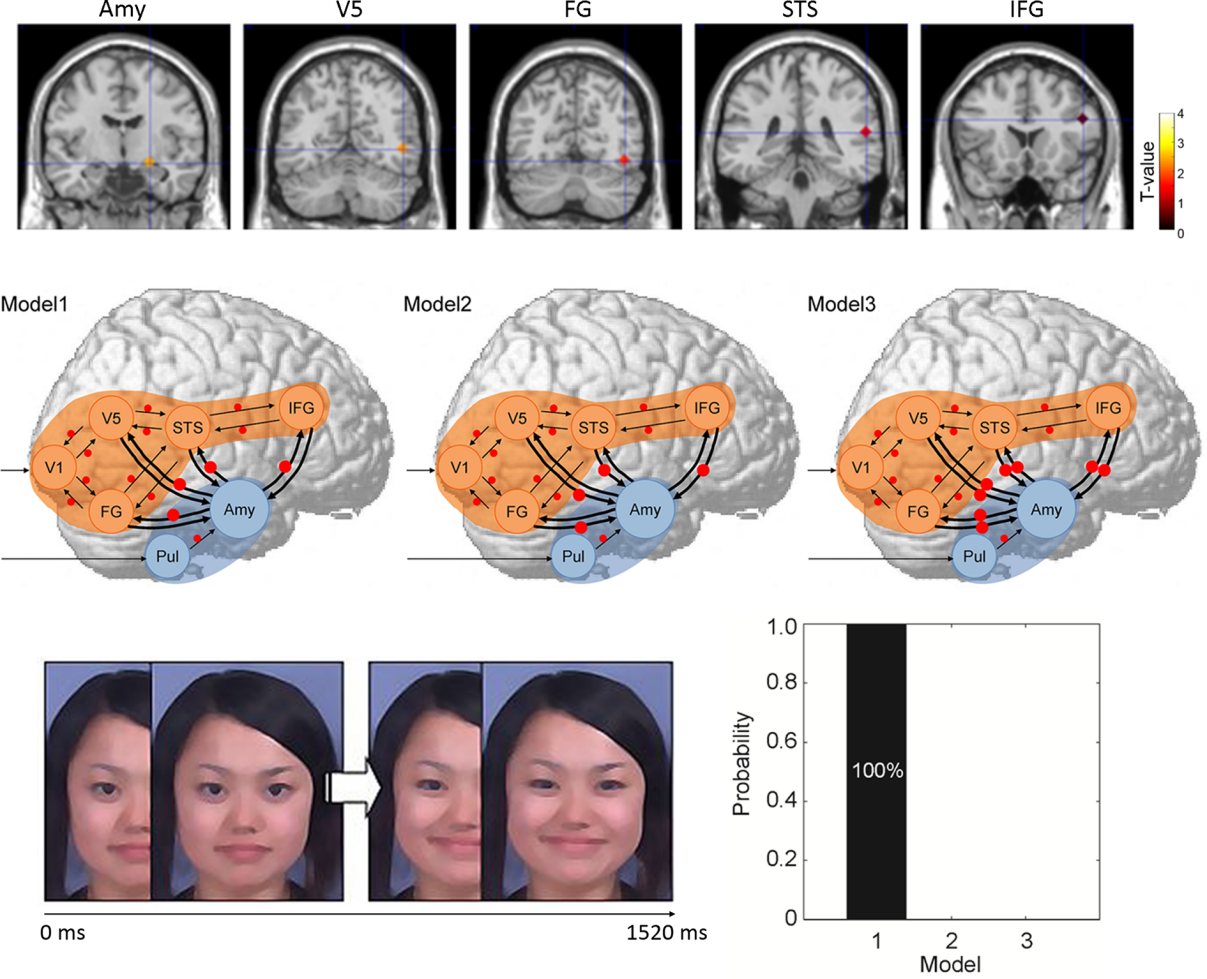
Study by Sato et al. (45). (Upper) Regions of interest in terms of regional brain activity and dynamic causal modeling analysis rendered on the spatially normalized brain of a representative participant. The degree of activation (a stronger response to dynamic facial expressions vs. dynamic mosaics in typically developing (TD) vs. autism spectrum disorder (ASD) groups) is overlaid using a red-yellow color scale. (Middle) Models for differences between ASD and TD groups in terms of the modulatory effect of dynamic expression in dynamic causal modeling. Arrows indicate intrinsic connections between brain regions. Red circles indicate modulation of dynamic expression. (Lower left) Illustration of dynamic facial expression. (Lower right) Results of model comparison for group differences. Amy, amygdala; FG, fusiform gyrus; IFG, inferior frontal gyrus; STS, superior temporal sulcus; V5, fifth visual area/middle temporal area.

The dynamic expression stimuli used were video clips of Japanese models that progressed from neutral to angry or from neutral to happy in terms of facial expression (**Figure 2**). Dynamic randomized mosaic images were created using the same materials. All dynamic expression frames were divided into very small squares and randomly re-ordered to yield dynamic information lacking facial features. Corresponding to the original dynamic expressions, the mosaic image stimuli were serially presented as a moving clip.

During each stimulus trial, after point fixation, the dynamic face or dynamic mosaic stimulus was presented. During each dummy-target trial, a red cross was presented instead of the stimulus and participants were instructed to press a button when the cross appeared.

Image scanning was performed with the aid of a 3-T scanning system at ATR Brain Activity Imaging Center. Image analyses (**Supplementary Figure 1**) featured standard preprocessing procedures and statistical tests at the group level (62). For data analyses, first region of interest (ROI) analyses were conducted using multivariate analyses of covariance and follow-up univariate *t*-tests. Then, DCM was performed in a standard manner (63–65). To investigate the direction of functional interaction between the amygdala and neocortex, seven brain regions (the pulvinar, amygdala, primary visual cortex, fifth visual area, fusiform gyrus, superior temporal sulcus, and inferior frontal gyrus) of the right hemisphere were selected (**Figure 2**). The coordinates of each ROI were derived from the results of a previous study (58). We assumed that the neocortical, social signal processing, network was in play based on previous evidence (66, 67). We also assumed that the subcortical visual pathway to the amygdala processed emotional facial expressions based on previous evidence (68, 69). Three models were constructed in terms of the direction of the modulatory effects (i.e., change in the connectivity between regions caused by experimental manipulation) of dynamic facial expressions: from the amygdala to the neocortex, from the neocortex to the amygdala, and bidirectional (**Figure 2**). We compared these models and selected that which optimally explained the data (70). Between-group differences in the modulatory effects of dynamic expressions were also evaluated (71).

#### Results

ROI analyses revealed that activities in the predefined brain regions during observing dynamic facial expressions (compared with dynamic mosaics) were significantly lower in the ASD than the TD group.

We used DCM to compare three models of the modulatory influences of dynamic facial expressions between the amygdala and neocortical network (**Figure 2**). This revealed that modulation of dynamic facial expressions from the amygdala to the neocortical network best explained the differences between the ASD and TD groups (**Figure 2**). The profile of modulatory effects demonstrated that modulation of dynamic expressions was reduced in all connections from the amygdala to the neocortical regions in the ASD group.

#### Discussion

The overall activities of the subcortical and neocortical regions involved in specific aspects of facial expression processing (e.g., the amygdala) were reduced in the ASD group during observation of dynamic facial expressions. These results are largely consistent with previous findings (46, 72).

Furthermore, DCM demonstrated that the model featuring modulation of dynamic facial expression from the amygdala to the neocortical network optimally explained the differences between the ASD and TD groups. The ASD group exhibited reduced modulation. The atypical functional networking patterns in individuals with ASD are in line with the previous description of weak modulation of dynamic expression within neocortical regions (72) and weak resting-state connectivity between the amygdala and neocortical regions (73) in ASD groups. However, this is the first evidence that modulation from the amygdala to the neocortex is weak in individuals with ASD during dynamic facial expression processing.

### Neurocognitive Model of Weak Amygdala’s Modulation in Autism

In summary, our psychological data demonstrate that individuals with ASD exhibit impaired rapid emotional modulation of attentional shift triggered by gaze. Our fMRI study demonstrated that individuals with ASD exhibit weak modulation from the amygdala to the neocortex during facial expression processing.

Although there is minimal direct evidence in individuals with ASD related to these topics, findings and theories in TD individuals have provided suggestions to further understand these psychological and neural phenomena. From a psychological perspective, considerable evidence and influential theories suggest that emotional processing can be rapidly implemented, even before subjective stimulus perception (74). Abundant evidence and theories suggest that emotional processing can modulate various types of other cognitive and motor processes (75, 76). Furthermore, psychological theories of emotion suggest that emotional processing is conducted for each external stimulus regardless of clear subjective experience (77). These data suggest that rapid emotional modulation of other processes is important in social interaction.

From a neural perspective, previous functional neuroimaging studies in TD participants have revealed that the amygdala implements emotional processing of external stimuli, even before conscious perception of the stimuli, *via* subcortical visual pathways (68, 78). Electrophysiological studies directly recording electric amygdala activity demonstrated that emotion-related activity occurs in the amygdala within 100 ms (79). Earlier DCM of electrophysiological data from TD participants suggested that modulation of dynamic facial expressions from the amygdala to the neocortical network commences before 200 ms (58). Other electrophysiological studies with TD individuals also showed that the emotional modulation of the neocortex during facial expression processing begins at approximately 200 ms (80, 81). Collectively, these data suggest that the amygdala rapidly modulates activity in the neocortex *via* the subcortical visual pathway, in accordance with the emotional significance of stimuli.

**Figure 3:**
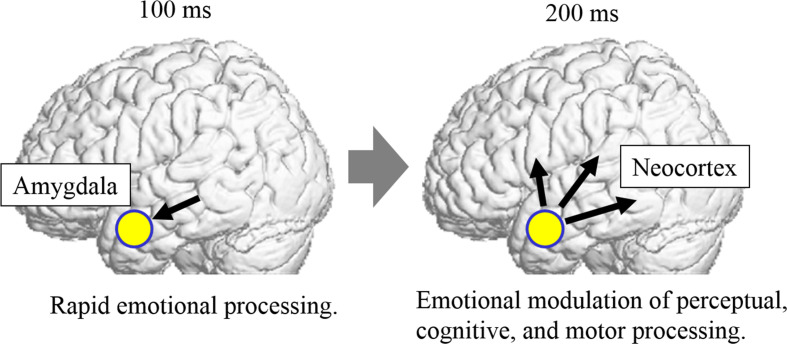
Hypothetical neurocognitive mechanisms underlying social atypicalities of autism spectrum disorder (ASD). In this model, individuals with ASD exhibit weak emotion-related activation in the amygdala *via* the subcortical visual pathway at approximately 100 ms (left). They also exhibit weak modulation from the amygdala to the widespread neocortical network, which is associated with modulated perceptual, cognitive, and motor processing functions of social stimuli, at approximately 200 ms (right).

Based on our results, together with these data, we formulate a hypothesis regarding the neurocognitive mechanisms underlying social atypicalities of ASD (**Figure 3**). From a psychological perspective, individuals with ASD have weak implementation of [1] rapid emotional processing and [2] emotional modulation of diverse processing of social stimuli. From a neural perspective, individuals with ASD have reductions in [1] rapid emotion-related amygdala activity *via* the subcortical visual pathway at approximately 100 ms and [2] emotion-related modulation from the amygdala to the widespread neocortical network at approximately 200 ms, which are associated with modulated perceptual, cognitive, and motor processing of social stimuli. This hypothesis can integrate previous psychological and neuroscientific findings of atypicalities in divergent psychological and neural processing in individuals with ASD, as described in the Introduction.

We also hypothesize that the neurocognitive mechanisms of weak amygdala modulation may be important during early development in individuals with ASD. Researchers have proposed that subcortical regions functioning from early infancy, such as the amygdala, show a bias to face- and eye-like stimuli; these regions develop cortical specialization for face and gaze processing through modulation of cortical activity (82). An anatomical study with large samples identified the smaller volume of subcortical regions (e.g., the amygdala) throughout development in individuals with ASD compared with TD individuals (56). However, it is unclear whether infants later diagnosed with autism initially exhibit a lack of bias to face and direct gaze (83). Investigation of the response to emotional facial expressions might be a promising approach to more effectively differentiate typical infants from infants with high risk of autism, because emotional facial expression triggers a robust activation of the amygdala (84); moreover, emotional sharing during joint attention facilitates the development of social functions (85). There have been few studies regarding this perspective in early infancy, although a recent study demonstrated a comparable attentional bias to fearful facial expressions in infants at low and high risks of ASD (86). To confirm the hypothesis of weak amygdala modulation in response to social stimuli, future studies should explore the processing of emotional facial expressions and the underlying neural correlates in individuals with ASD from infancy to adulthood.

Our hypothesis positing weak amygdala’s modulation of the widespread neocortical regions may have practical implications, suggesting that improved amygdala activity might exert several positive effects on perceptual, cognitive, or motor processing of social stimuli. Consistent with this notion, a previous study has shown that electrical stimulation of the amygdala in individuals with ASD modified their autistic symptoms and face-to-face interaction behaviors (87). The influence of oxytocin may also be relevant. Some previous psychological studies have shown that intranasal administration of oxytocin to individuals with ASD facilitated their perceptual and cognitive processing of emotional facial expressions (88, 89). An fMRI study demonstrated that improved emotion recognition based on facial expressions due to intranasal administration of oxytocin was associated with increased amygdala reactivity in response to facial stimuli in ASD and TD participants (90). Modulation from the amygdala to the neocortical regions may thus explain the behavioral influences of oxytocin in individuals with ASD, although the effect of oxytocin on social atypicalities in ASD remains controversial (91). In sum, electrical or pharmacological intervention to modulate the activity of the amygdala may usefully improve social functioning in individuals with ASD.

However, it must be noted that several issues remain, and future extension of this work is needed. First, because the hypothetical model was proposed based on relatively few findings, more evidence is needed, especially neuroscientific evidence. Because psychological studies have demonstrated atypical emotional modulation of social stimuli in individuals with ASD using various types of psychological tasks (e.g., the cueing paradigm), neuroimaging investigation using such tasks could provide direct tests of the neurocognitive model we proposed. Testing different stimuli (e.g., movies and live interactions) would be valuable, as previous fMRI studies have reported different activation patterns with different stimuli; for example, static social stimuli evoked different patterns of neural activity compared to dynamic stimuli in individuals with ASD (46, 72).

Second, because findings regarding ASD could be subject to multiple confounding factors, such as trait anxiety (92) and alexithymia (93), which could be associated atypical amygdala function (94, 95), further investigations would be needed to separate the effects of core ASD symptoms from the effects of these factors on psychological and neural activity in the proposed model.

Third, the neurocognitive model we propose in this article may not be specific to ASD but common across several neurodevelopmental and neuropsychiatric disorders. Some studies have reported behavioral problems having to do with emotional modulation and neurological abnormalities in amygdala activity and connectivity in other clinical groups. For example, previous studies have reported that individuals with attention-deficit hyperactivity disorder were impaired in emotion recognition from facial expressions (96) and showed atypical amygdala activity (97). Both the sensitivity and specificity of the model in describing the social atypicalities of ASD should be tested.

## Conclusions

Based on our psychological and neuroimaging data and other related evidence, we presented our hypothetical neurocognitive model that weak modulation from the amygdala to neocortical regions—through which emotion rapidly modulates various types of perceptual, cognitive, and motor processing functions—underlies social atypicalities in individuals with ASD. Our model may enable integration of divergent psychological and neuroscientific findings showing atypical psychological and neural processes in individuals with ASD. However, the model lacks sufficient evidence. We hope that our hypothesis will stimulate empirical research regarding social atypicalities in individuals with ASD.

## Data Availability Statement

The original contributions presented in the study are included in the article/supplementary material; further inquiries can be directed to the corresponding author.

## Ethics Statement

Written informed consent was obtained from the individual(s) for the publication of any potentially identifiable images or data included in this article.

## Author Contributions

All authors designed the research and wrote the manuscript. All authors contributed to the article and approved the submitted version.

## Funding

This study was supported by funds from Japan Science and Technology Agency CREST (JPMJCR17A5).

## Conflict of Interest

TK was employed by company ATR-Promotions.

The remaining authors declare that the research was conducted in the absence of any commercial or financial relationships that could be construed as a potential conflict of interest.

## Supplementary Material

Supplementary Figure 1 Flowchart of functional magnetic resonance imaging (fMRI) data analysis. The fMRI data for each subject were first preprocessed (realigned using the first scan as a reference to correct for head motion, corrected for slice timing, coregistered to the anatomical image, normalized to the Montreal Neurological Institute space, and smoothed with an isotopic Gaussian kernel). Next, a general linear model was estimated for each subject. For regional brain activity analyses, multivariate analyses of covariance were conducted on the beta estimates (i.e., task-related increments of each subject’s blood oxygen level-dependent signal) for regions of interest. For effective or directional connectivity analysis, dynamic causal modeling (DCM) was performed on three network models dealing with the amygdala-neocortex interaction using time series data extracted from the regions of interest of individual subjects. Group level DCMs were estimated using a second-level parametric empirical Bayesian DCM engine. Bayesian model comparisons and parameter inferences derived *via* Bayesian averaging were accomplished to evaluate group differences.

## References

[1] American Psychiatric Association Diagnostic and statistical manual of mental disorders. 5th ed Arlington (VA): American Psychiatric Publishing (2013).

[2] Hobson RP Autism and the development of mind. Lawrence Erlbaum Associates: Hove (UK) (1993).

[3] Uljarevic M Hamilton A Recognition of emotions in autism: a formal metaanalysis. J Autism Dev Disord (2013) 43(7):1517–26. 10.1007/s10803-012-1695-5 23114566

[4] Tang J Falkmer M Horlin C Tan T Vaz S Falkmer T Face recognition and visual search strategies in autism spectrum disorders: Amending and extending a recent review by Weigelt et al. PloS One (2015) 10:e0134439. 10.1371/journal.pone.0134439 26252877 PMC4529109

[5] Mundy P Sigman M Kasari C Understanding other minds: Perspectives from autism. In: . The theory of mind and joint-attention deficits in autism. New York (NY): Oxford University Press (1994). p. 181–203.

[6] Canigueral R Hamilton AFC The role of eye gaze during natural social interactions in typical and autistic. People Front Psychol (2019) 10:560. 10.3389/fpsyg.2019.00560 30930822 PMC6428744

[7] Tanaka JW Sung A The “eye avoidance” hypothesis of autism face processing. J Autism Dev Disord (2016) 46(5):1538–52. 10.1007/s10803-013-1976-7 PMC3997654 24150885

[8] Williams JH Whiten A Singh T A systematic review of action imitation in autistic spectrum disorder. J Autism Dev Disord (2004) 34:285–99. 10.1023/B:JADD.0000029551.56735.3a 15264497

[9] Federici A Parma V Vicovaro M Radassao L Casartelli L Ronconi L Anomalous perception of biological motion in autism: A conceptual review and meta-analysis. Sci Rep (2020) 10:4576. 10.1038/s41598-020-61252-3 32165647 PMC7067769

[10] Nadel J perception-action coupling and imitation in autism spectrum disorder. Dev Med Child Neurol (2015) 57:55–8. 10.1111/dmcn.12689 25690119

[11] Baron-Cohen S Mindblindness: An essay on autism and theory of mind. Cambridge (MA): MIT Press (1995).

[12] Brewer N Young RL Barnett E Measuring theory of mind in adults with autism spectrum disorder. J Autism Dev Disord (2017) 47:1927–41. 10.1007/s10803-017-3080-x PMC5487761 28275927

[13] Livingston LA Carr B Shah P Recent advances and new directions in measuring theory of mind in autistic adults. J Autism Dev Disord (2019) 49:1738–44. 10.1007/s10803-018-3823-3 PMC6450842 30515619

[14] Amaral DG Dawson G Geschwind DH Autism spectrum disorders. New York (NY): Oxford University Press (2011).

[15] Sato W Uono S The atypical social brain network in autism: advances in structural and functional MRI studies. Curr Opin Neurol (2019) 32(4):617–21. 10.1097/WCO.0000000000000713 31135458

[16] Bernhardt BC Di Martino A Valk SL Wallace GL Neuroimaging-based phenotyping of the autism spectrum. Curr Top Behav Neurosci (2017) 30:341–55. 10.1007/7854_2016_438 26946501

[17] Müler RA Fishman I Brain connectivity and neuroimaging of social networks in autism. Trends Cognit Sci (2018) 22:1103–16. 10.1016/j.tics.2018.09.008 PMC7080636 30391214

[18] Uono S Sato W Toichi M Dynamic fearful gaze does not enhance attention orienting in individuals with Asperger’s disorder. Brain Cogn (2009) 71(3):229–33. 10.1016/j.bandc.2009.08.015 19781841

[19] Mundy P Sigman M Ungerer J Sherman T Defining the social deficits of autism: The contribution of nonverbal communication measures. J Child Psychol Psychiatry (1986) 27(5):657–69. 10.1111/j.1469-7610.1986.tb00190.x 3771682

[20] Posner MI Orienting of attention. Q J Exp Psychol (1980) 32(1):3–25. 10.1080/00335558008248231 7367577

[21] Okada T Sato W Murai T Kubota Y Toichi M Eye gaze triggers visuospatial attentional shift in individuals with autism. Psychologia (2003) 46(4):246–54. 10.2117/psysoc.2003.246

[22] Graham R Friesen CK Fichtenholz HM LaBar KS Modulation of reflexive orienting to gaze direction by facial expressions. Vis Cogn (2010) 18:331–68. 10.1080/13506280802689281

[23] Lassalle A Itier RJ Fearful, surprised, happy, and angry facial expressions modulate gaze-oriented attention: Behavioral and ERP evidence. Soc Neurosci (2013) 8:583–600. 10.1080/17470919.2013.835750 24047232 PMC3925118

[24] Lassalle A Itier RJ Emotional modulation of attention orienting by gaze varies with dynamic cue sequence. Vis Cogn (2015) 23:720–35. 10.1080/13506285.2015.1083067 PMC5362272 28344502

[25] Liu J Shi Y Whitaker L Tian Y Hu Z Facial expressions modulate the gaze orienting effect on sound localization judgement. Vis Cogn (2019) 27:109–19. 10.1080/13506285.2019.1606128

[26] McCrackin SD Itier RJ Both fearful and happy expressions interact with gaze direction by 200 ms SOA to speed attention orienting. Vis Cogn (2018) 26:231–52. 10.1080/13506285.2017.1420118

[27] McCrackin SD Itier RJ Individual differences in the emotional modulation of gaze-cuing. Cognit Emot (2019) 33:768–800. 10.1080/02699931.2018.1495618 29983094

[28] Neath K Nilsen ES Gittsovich K Itier RJ Attention orienting by gaze and facial expressions across development. Emotion (2013) 13:397–408. 10.1037/a0030463 23356559 PMC3925116

[29] Uono S Sato W Toichi M Dynamic fearful expressions enhance gaze-triggered attention orienting in high and low anxiety individuals. Soc Behav Pers (2009) 37:1313–26. 10.2224/sbp.2009.37.10.1313

[30] Dalmaso M Castelli L Galfano G Social modulators of gaze-mediated orienting of attention: a review. Psychon Bull Rev (in press). 10.3758/s13423-020-01730-x 32291650

[31] Lassalle A Itier RJ Autistic traits influence gaze-oriented attention to happy but not fearful faces. Soc Neurosci (2015) 10:70–88. 10.1080/17470919.2014.958616 25222883 PMC5325724

[32] Uono S Sato W Toichi M The specific impairment of fearful expression recognition and its atypical development in pervasive developmental disorder. Soc Neurosci (2011) 6(5–6):452–63. 10.1080/17470919.2011.605593 21919566

[33] Hall GB West CD Szatmari P Backward masking: Evidence of reduced subcortical amygdala engagement in autism. Brain Cogn (2007) 65(1):100–6. 10.1016/j.bandc.2007.01.010 17629385

[34] Akechi H Senju A Kikuchi Y Tojo Y Osanai H Hasegawa T Does gaze direction modulate facial expression processing in children with autism spectrum disorder? Child Dev (2009) 80(4):1134–46. 10.1111/j.1467-8624.2009.01321.x 19630898

[35] Akechi H Senju A Kikuchi Y Tojo Y Osanai H Hasegawa T The effect of gaze direction on the processing of facial expressions in children with autism spectrum disorder: An ERP study. Neuropsychologia (2010) 48(10):2841–51. 10.1016/j.neuropsychologia.2010.05.026 20546762

[36] Ioannou C Shared mechanism for emotion processing in adolescents with and without autism. Sci Rep (2017) 7:42696. 10.1038/srep42696 28218248 PMC5317002

[37] Tell D Davidson D Camras LA Recognition of emotion from facial expressions with direct or averted eye gaze and varying expression intensities in children with autism disorder and typically developing children. Autism Res Treat (2014) 2014:816137. 10.1155/2014/816137 24804098 PMC3996291

[38] Vida MD Maurer D Calder AJ Rhodes G Walsh JA Pachai MV The influences of face inversion and facial expression on sensitivity to eye contact in high-functioning adults with autism spectrum disorders. Autism Dev Disord (2013) 43:2536–48. 10.1007/s10803-013-1802-2 23471478

[39] Ekman P Friesen WV Pictures of Facial Affect. Palo Alto (CA): Consulting Psychologist (1976).

[40] Bayliss AP Frischen A Fenske MJ Tipper SP Affective evaluations of objects are influenced by observed gaze direction and emotional expression. Cognition (2007) 104(3):644–53. 10.1016/j.cognition.2006.07.012 16950239

[41] Shamay-Tsoory SG Tibi-Elhanany Y Aharon-Peretz J The green-eyed monster and malicious joy: the neuroanatomical bases of envy and gloating (schadenfreude). Brain (2007) 130(6):1663–78. 10.1093/brain/awm093 17525143

[42] Sato W Sawada R Uono S Yoshimura S Kochiyama T Kubota Y Impaired detection of happy facial expressions in autism. Sci Rep (2017) 7(1):13340. 10.1038/s41598-017-11900-y 29042592 PMC5645383

[43] Uono S Sato W Toichi M Reduced representational momentum for subtle dynamic facial expressions in individuals with autism spectrum disorders. Res Autism Spectr Disord (2014) 8(9):1090–9. 10.1016/j.rasd.2014.05.018

[44] Yoshimura S Sato W Uono S Toichi M Impaired overt facial mimicry in response to dynamic facial expressions in high-functioning autism spectrum disorders. J Autism Dev Disord (2015) 45(5):1318–28. 10.1007/s10803-014-2291-7 25374131

[45] Sato W Kochiyama T Uono S Yoshimura S Kubota Y Sawada R Atypical amygdala–Neocortex interaction during dynamic facial expression processing in autism spectrum disorder. Front Hum Neurosci (2019) 13:351. 10.3389/fnhum.2019.00351 31680906 PMC6813184

[46] Pelphrey KA Morris JP McCarthy G Labar KS Perception of dynamic changes in facial affect and identity in autism. Soc Cognit Affect Neurosci (2007) 2(2):140–9. 10.1093/scan/nsm010 PMC2174259 18174910

[47] Calder AJ Lawrence AD Young AW Neuropsychology of fear and loathing. Nat Rev Neurosci (2001) 2(5):352–63. 10.1038/35072584 11331919

[48] Haxby JV Hoffman EA Gobbini MI The distributed human neural system for face perception. Trends Cognit Sci (2000) 4(6):223–33. 10.1016/S1364-6613(00)01482-0 10827445

[49] Rizzolatti G Fogassi L Gallese V Neurophysiological mechanisms underlying the understanding and imitation of action. Nat Rev Neurosci (2001) 2(9):661–70. 10.1038/35090060 11533734

[50] Bickart KC Dickerson BC Barrett LF The amygdala as a hub in brain networks that support social life. Neuropsychologia (2014) 63:235–48. 10.1016/j.neuropsychologia.2014.08.013 PMC4981504 25152530

[51] Bachevailer J The amygdala, social behaviour, and autism. In: Aggleton JP, editor. The Amygdala: A Functional Analysis. New York, NY: Oxford University Press (2000). p. 509–44.

[52] Amaral DG Bauman MD Schumann CM The amygdala and autism: implications from non-human primate studies. Genes Brain Behav (2003) 2(5):295–302. 10.1034/j.1601-183X.2003.00043.x 14606694

[53] Kemper TL Bauman ML The contribution of neuropathologic studies to the understanding of autism. Neurol Clinics (1993) 11:175–87. 10.1016/S0733-8619(18)30176-2 8441369

[54] Schumann CM Amaral DG Stereological analysis of amygdala neuron number in autism. J Neurosci (2006) 26:7674–9. 10.1523/JNEUROSCI.1285-06.2006 PMC6674270 16855095

[55] Aylward EH Minshew NJ Goldstein G Honeycutt NA Augustine AM Yates KO MRI volumes of amygdala and hippocampus in non–mentally retarded autistic adolescents and adults. Neurology (1999) 53:2145–50. 10.1212/WNL.53.9.2145 10599796

[56] van Rooij D Anagnostou E Arango C Auzias G Behrmann M Busatto GF Cortical and subcortical brain morphometry differences between patients with autism spectrum disorders (ASD) and healthy individuals across the lifespan: results from the ENIGMA-ASD working group. Am J Psychiatry (2018) 175(4):359–69. 10.1176/appi.ajp.2017.17010100 PMC6546164 29145754

[57] Gibbard CR Ren J Skuse DH Clayden JD Clark CA Structural connectivity of the amygdala in young adults with autism spectrum disorder. Hum Brain Mapp (2018) 39:1270–82. 10.1002/hbm.23915 PMC5838552 29265723

[58] Sato W Kochiyama T Uono S Yoshikawa S Toichi M Direction of amygdala–neocortex interaction during dynamic facial expression processing. Cereb Cortex (2017) 27(3):1878–90. 10.1093/cercor/bhw036 26908633

[59] Friston KJ Harrison L Penny W Dynamic causal modelling. Neuroimage (2003) 19(4):1273–302. 10.1016/S1053-8119(03)00202-7 12948688

[60] Day-Brown JD Wei H Chomsung RD Petry HM Bickford ME Pulvinar projections to the striatum and amygdala in the tree shrew. Front Neuroanat (2010) 4:143. 10.3389/fnana.2010.00143 21120139 PMC2991220

[61] Amaral DG Price JL Pitkanen A Pitkanen A Carmichael ST The amygdala: Neurobiological aspects of emotion, memory, and mental dysfunction. In: . Anatomical organization of the primate amygdaloid complex. New York (NY): Oxford University Press (1992). p. 1–66.

[62] Holmes AP Friston KJ Generalisability, random effects and population inference. Neuroimage (1998) 7:S754. 10.1016/S1053-8119(18)31587-8

[63] Friston KJ Litvak V Oswal A Razi A Stephan KE van Wijk BCM Bayesian model reduction and empirical Bayes for group (DCM) studies. Neuroimage (2016) 128:413–31. 10.1016/j.neuroimage.2015.11.015 PMC4767224 26569570

[64] Zeidman P Jafarian A Seghier ML Litvak V Cagnan H Price CJ A guide to group effective connectivity analysis, part 2: second level analysis with PEB. Neuroimage (2019) 200:12–25. 10.1016/j.neuroimage.2019.06.032 31226492 PMC6711451

[65] Zeidman P Jafarian A Corbin N Seghier ML Razi A Price CJ A guide to group effective connectivity analysis, part 1: first level analysis with DCM for fMRI. Neuroimage (2019) 200:174–90. 10.1016/j.neuroimage.2019.06.031 PMC6711459 31226497

[66] Oram MW Perrett DI Integration of form and motion in the anterior superior temporal polysensory area (STPa) of the macaque monkey. J Neurophysiol (1996) 76(1):109–29. 10.1152/jn.1996.76.1.109 8836213

[67] Hamilton AF Emulation and mimicry for social interaction: A theoretical approach to imitation in autism. Q J Exp Psychol (2008) 61(1):101–15. 10.1080/17470210701508798 18038342

[68] Vuilleumier P How brains beware: Neural mechanisms of emotional attention. Trends Cognit Sci (2005) 9(12):585–94. 10.1016/j.tics.2005.10.011 16289871

[69] Morris JS Ohman A Dolan RJ A subcortical pathway to the right amygdala mediating “unseen” fear. Proc Natl Acad Sci U S A (1999) 96(4):1680–5. 10.1073/pnas.96.4.1680 PMC15559 9990084

[70] Stephan KE Penny WD Daunizeau J Moran RJ Friston KJ Bayesian model selection for group studies. Neuroimage (2009) 46(4):1004–17. 10.1016/j.neuroimage.2009.03.025 PMC2703732 19306932

[71] Penny WD Stephan KE Daunizeau J Rosa MJ Friston KJ Schofield TM Comparing families of dynamic causal models. PloS Comput Biol (2010) 6:e1000709. 10.1371/journal.pcbi.1000709 20300649 PMC2837394

[72] Sato W Toichi M Uono S Kochiyama T Impaired social brain network for processing dynamic facial expressions in autism spectrum disorders. BMC Neurosci (2012) 13:99. 10.1186/1471-2202-13-99 22889284 PMC3459703

[73] Rausch A Zhang W Haak KV Mennes M Hermans EJ van Oort E Altered functional connectivity of the amygdaloid input nuclei in adolescents and young adults with autism spectrum disorder: a resting state fMRI study. Mol Autism (2016) 7:13. 10.1186/s13229-015-0060-x 26823966 PMC4730628

[74] Zajonc RB On the primacy of affect. Am Psychol (1984) 39(2):117–23. 10.1037/0003-066X.39.2.117

[75] Levenson RW The intrapersonal functions of emotion. Cognit Emot (1999) 13:481–504. 10.1080/026999399379159

[76] Oatley K Johnson-Laird PN Striving and feeling: Interactions among goals, affect, and self-regulation. In: The communicative theory of emotions: Empirical tests, mental models, and implications for social interaction. Hillsdale (NJ): Lawrence Erlbaum Associates (1996). p. 363–93.

[77] Wundt W An introduction to psychology. London: G. Allen (1912). p. 1–198.

[78] Sato W Kochiyama T Minemoto K Sawada R Fushiki T Amygdala activation during unconscious visual processing of food. Sci Rep (2019) 9(1):7277. 10.1038/s41598-019-43733-2 31086241 PMC6513994

[79] Sato W Kochiyama T Uono S Matsuda K Usui K Inoue Y Rapid amygdala gamma oscillations in response to fearful facial expressions. Neuropsychologia (2011) 49(4):612–7. 10.1016/j.neuropsychologia.2010.12.025 21182851

[80] Balconi M Pozzoli U Face-selective processing and the effect of pleasant and unpleasant emotional expressions on ERP correlates. Int J Psychophysiol (2003) 49(1):67–74. 10.1016/S0167-8760(03)00081-3 12853131

[81] Sato W Kochiyama T Yoshikawa S Matsumura M Emotional expression boosts early visual processing of the face: ERP recording and its decomposition by independent component analysis. Neuroreport (2001) 12(4):709–14. 10.1097/00001756-200103260-00019 11277569

[82] Johnson MH Senju A Tomalski P The two-process theory of face processing: modifications based on two decades of data from infants and adults. Neurosci Biobehav Rev (2015) 50:169–79. 10.1016/j.neubiorev.2014.10.009 25454353

[83] Guillon Q Hadjikhani N Baduel S Rogé B Visual social attention in autism spectrum disorder: insights from eye tracking studies. Neurosci Biobehav Rev (2014) 42:279–97. 10.1016/j.neubiorev.2014.03.013 24694721

[84] Fusar-Poli P Placentino A Carletti F Landi P Allen P Surguladze S Functional atlas of emotional faces processing: a voxel-based meta-analysis of 105 functional magnetic resonance imaging studies. J Psychiatry Neurosci (2009) 34(6):418–32. PMC2783433 19949718

[85] Mundy P Sigman M The theoretical implications of joint-attention deficits in autism. Dev Psychopath (1989) 1(3):173–83. 10.1017/S0954579400000365

[86] Wagner JB Keehn B Tager-Flusberg H Nelson CA Attentional bias to fearful faces in infants at high risk for autism spectrum disorder. Emotion (in press). 10.1037/emo0000628 PMC6986980 31355652

[87] Sturm V Fricke O Bührle CP Lenartz D Maarouf M Treuer H DBS in the basolateral amygdala improves symptoms of autism and related self-injurious behavior: A case report and hypothesis on the pathogenesis of the disorder. Front Hum Neurosci (2013) 6:341. 10.3389/fnhum.2012.00341 23346052 PMC3549527

[88] Domes G Normann C Heinrichs M The effect of oxytocin on attention to angry and happy faces in chronic depression. BMC Psychiatry (2016) 16:92. 10.1186/s12888-016-0794-9 27048333 PMC4822232

[89] Xu L Ma X Zhao W Luo L Yao S Kendrick KM Oxytocin enhances attentional bias for neutral and positive expression faces in individuals with higher autistic traits. Psychoneuroendocrinology (2015) 62:352–8. 10.1016/j.psyneuen.2015.09.002 26372768

[90] Domes G Kumbier E Heinrichs M Herpertz SC Oxytocin promotes facial emotion recognition and amygdala reactivity in adults with asperger syndrome. Neuropsychopharmacology (2014) 39:698–706. 10.1038/npp.2013.254 24067301 PMC3895247

[91] Keech B Crowe S Hocking DR Intranasal oxytocin, social cognition and neurodevelopmental disorders: A meta-analysis. Psychoneuroendocrinology (2018) 87:9–19. 10.1016/j.psyneuen.2017.09.022 29032324

[92] White SW Bray BC Ollendick TH Examining shared and unique aspects of social anxiety disorder and autism spectrum disorder using factor analysis. J Autism Dev Disord (2012) 42:874–84. 10.1007/s10803-011-1325-7 21713589

[93] Berthoz S Hill EL The validity of using self-reports to assess emotion regulation abilities in adults with autism spectrum disorder. Europ Psychiatry (2005) 20:291–8. 10.1016/j.eurpsy.2004.06.013 15935431

[94] Reker M Ohrmann P Rauch AV Kugel H Bauer J Dannlowski U Individual differences in alexithymia and brain response to masked emotion faces. Cortex (2010) 46(5):658–67. 10.1016/j.cortex.2009.05.008 19524887

[95] Thomas KM Drevets WC Dahl RE Ryan ND Birmaher B Eccard CH Amygdala response to fearful faces in anxious and depressed children. Arch Gen Psychiatry (2001) 58(11):1057–63. 10.1001/archpsyc.58.11.1057 11695953

[96] Borhani K Nejati V Emotional face recognition in individuals withattention-deficit/hyperactivity disorder: A review article. Dev Neuropsychol (2018) 43(3):256–77. 10.1080/87565641.2018.1440295 29461118

[97] Brotman MA Rich BA Guyer AE Lunsford JR Horsey SE Reising MM Amygdala Activation During Emotion Processing of Neutral Faces in Children With Severe Mood Dysregulation Versus ADHD or Bipolar Disorder. Am J Psychiatry (2010) 167(1):61–9. 10.1176/appi.ajp.2009.09010043 PMC3075433 19917597

