# Supplementary figures and images for: Neurocognitive Mechanisms Underlying Social Atypicalities in Autism: Weak Amygdala’s Emotional Modulation Hypothesis
Source: Front Psychiatry. 2020 Sep 4;11:864. doi: 10.3389/fpsyt.2020.00864 (PMC7500257; doi:10.3389/fpsyt.2020.00864)

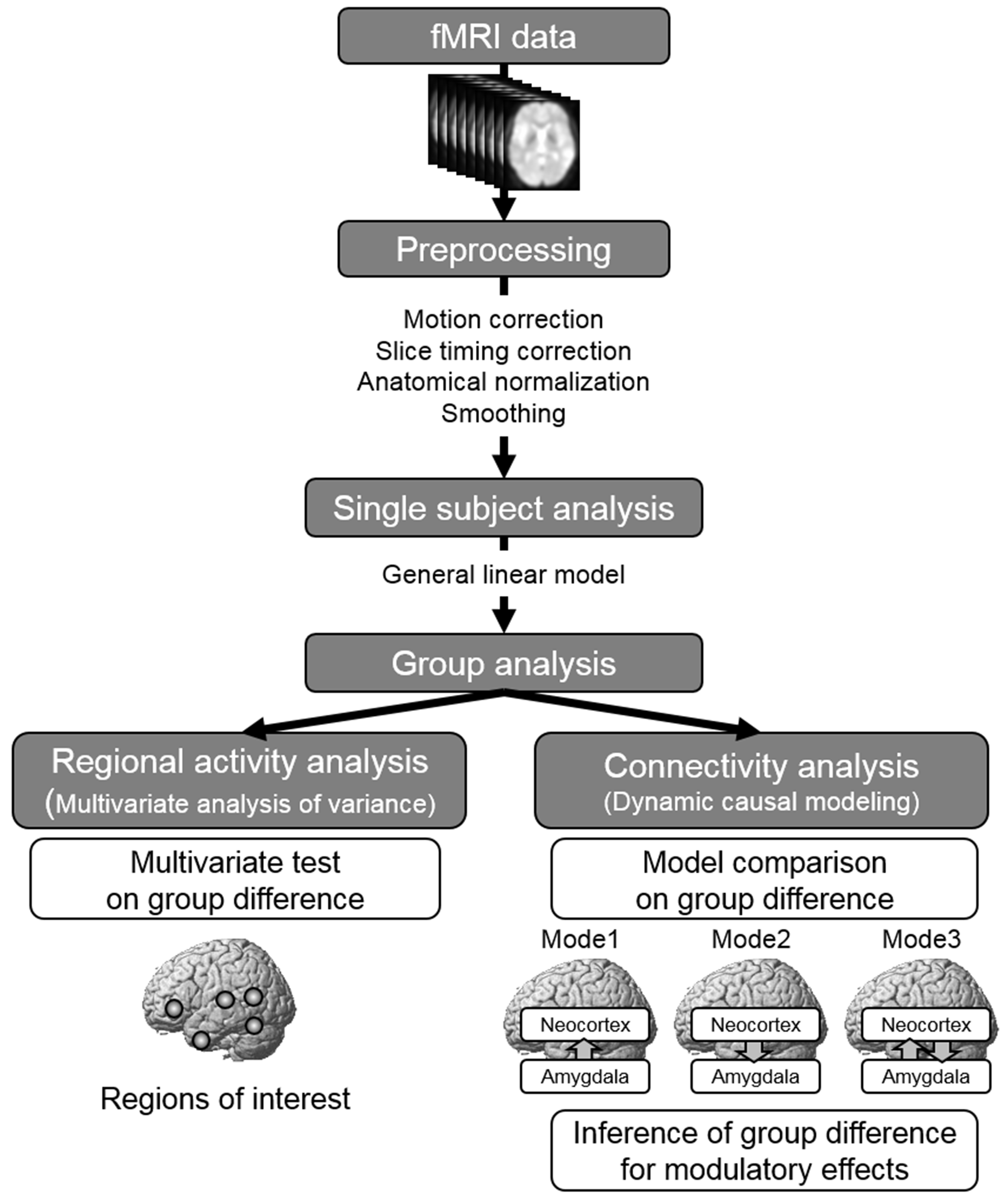

Supplement: Supplementary Figure 1 — Flowchart of functional magnetic resonance imaging (fMRI) data analysis. The fMRI data for each subject were first preprocessed (realigned using the first scan as a reference to correct for head motion, corrected for slice timing, coregistered to the anatomical image, normalized to the Montreal Neurological Institute space, and smoothed with an isotopic Gaussian kernel). Next, a general linear model was estimated for each subject. For regional brain activity analyses, multivariate analyses of covariance were conducted on the beta estimates (i.e., task-related increments of each subject’s blood oxygen level-dependent signal) for regions of interest. For effective or directional connectivity analysis, dynamic causal modeling (DCM) was performed on three network models dealing with the amygdala-neocortex interaction using time series data extracted from the regions of interest of individual subjects. Group level DCMs were estimated using a second-level parametric empirical Bayesian DCM engine. Bayesian model comparisons and parameter inferences derived via Bayesian averaging were accomplished to evaluate group differences. [file Image_1.tif]
